# The relationship between functional brain connectivity and neuroinflammatory processes—new insights into the pathomechanisms of ASD

**DOI:** 10.3389/fnins.2026.1787670

**Published:** 2026-03-23

**Authors:** Beata Zwierko, Alina Jaroch, Marietta Bracha, Bartosz Mruk, Jerzy Walecki

**Affiliations:** 1 Department of Electroradiology, Faculty of Health Sciences, Collegium Medicum in Bydgoszcz, Nicolaus Copernicus University in Torun, Bydgoszcz, Poland; 2 Division of Biochemistry and Biogerontology, Department of Geriatrics, Faculty of Health Sciences, Collegium Medicum in Bydgoszcz, Nicolaus Copernicus University in Torun, Bydgoszcz, Poland; 3 Center for Radiological Diagnostics, National Medical Institute of the Ministry of the Interior and Administration, Warsaw, Poland

**Keywords:** autism spectrum disorder, cytokines, functional connectivity, neuroinflammation, resting-state fMRI

## Abstract

Autism spectrum disorder (ASD) is a complex neurodevelopmental condition characterized by deficits in social communication and restricted, repetitive behaviors. Increasing evidence suggests that neuroinflammatory processes are closely associated with the pathophysiology of ASD, linking immune dysregulation with altered brain development and function. This review synthesizes current findings on the relationships between neuroinflammatory mechanisms, biochemical and metabolic alterations, and functional brain connectivity, as revealed by neuroimaging—particularly functional magnetic resonance imaging (fMRI). Across clinical, postmortem, and imaging studies, individuals with ASD show consistent evidence of microglial and astroglial activation, altered cytokine profiles (including IL-1β, IL-6, and TNF-α), and markers of oxidative stress such as glutathione imbalance and lipid peroxidation. These immune and metabolic alterations are associated with changes in synaptic plasticity, neurotransmission, and large-scale neuronal network organization, including altered functional connectivity within the default mode, salience, and executive control networks. Complementary imaging modalities further support links between glial activity, excitatory–inhibitory imbalance, and aberrant connectivity patterns. Emerging evidence also highlights interactions between inflammation, lipid metabolism, neurotransmitter systems (notably serotonin and dopamine), and genetic and epigenetic factors that modulate immune responses in ASD. Integrating inflammatory and metabolic biomarkers with fMRI and spectroscopic measures provides a promising framework for characterizing biologically informed ASD subtypes and advancing precision diagnostic and therapeutic strategies. Overall, current evidence supports a multilevel neuroimmune framework in which chronic inflammation and oxidative stress are associated with atypical functional brain connectivity in ASD. Future longitudinal and multimodal studies are required to validate candidate biomarkers, clarify mechanistic pathways, and evaluate interventions targeting neuroinflammatory processes.

## Introduction

In recent years, accumulating evidence has demonstrated that neuroinflammation plays a crucial role in the pathophysiology of autism spectrum disorder (ASD) (Matta et al., 2019; Estes and McAllister, 2015; Meltzer and Van de Water, 2017). ASD is a complex neurodevelopmental condition characterized by deficits in social communication, restricted interests, and repetitive behaviors. Although the etiology of ASD is multifactorial, numerous studies indicate that immune dysregulation and chronic low-grade inflammation are central mechanisms contributing to atypical brain development and function (Estes and McAllister, 2015; Meltzer and Van de Water, 2017).

Neuroinflammatory processes within the central nervous system (CNS) involve the activation of microglia and astrocytes and are accompanied by increased production of pro-inflammatory cytokines, including interleukin-6 (IL-6), tumor necrosis factor-α (TNF-α), and interleukin-1β (IL-1β) (Morgan et al., 2010; Chez et al., 2007). These mediators can profoundly influence synaptic maturation, neuronal connectivity, and neurochemical homeostasis (Bilbo and Schwarz, 2012). Imaging and postmortem studies have consistently demonstrated microglial activation, astroglial hypertrophy, and elevated cytokine expression in brain regions implicated in ASD, including the frontal cortex, cingulate gyrus, and cerebellum (Morgan et al., 2010). Such immune activation is thought to modulate synaptic plasticity and neurotransmission, leading to long-lasting alterations in neuronal circuit organization (Matta et al., 2019; Meltzer and Van de Water, 2017).

An increasing number of studies suggest that neuroinflammation directly affects the functional organization of the autistic brain. These alterations are increasingly captured by neuroimaging techniques, particularly functional magnetic resonance imaging (fMRI). Unlike structural or metabolic imaging approaches, fMRI enables the assessment of dynamic patterns of neuronal synchronization that reflect functional communication within and between large-scale neural networks. As such, fMRI provides a valuable framework for investigating how immune-mediated processes translate into altered brain function and behavior in individuals with ASD (Guo et al., 2024). Inflammatory mechanisms, including microglial and astroglial activation, may disrupt the balance between long-range and local connectivity, resulting in aberrant network synchronization (Zikopoulos and Barbas, 2013). This concept aligns with the functional disconnection hypothesis of ASD, which proposes that cognitive and social impairments arise from reduced integration across distributed cortical and subcortical systems (Just et al., 2012; Courchesne et al., 2019). Despite growing evidence linking neuroinflammation to both molecular abnormalities and altered functional connectivity in ASD, current findings remain fragmented across methodological approaches, and an integrative synthesis connecting inflammatory mechanisms with large-scale network dysfunction is still lacking.

Together, these observations support the view that neuroinflammation is not merely a secondary epiphenomenon but may contribute directly to atypical brain connectivity in ASD. Understanding how inflammatory pathways intersect with neurodevelopment and large-scale network organization provides a critical framework for identifying biomarkers and potential therapeutic targets. Therefore, the present literature review aims to synthesize current evidence linking neuroinflammatory processes with neurobiochemical and functional brain alterations in individuals with ASD. By integrating findings from clinical, immunological, and neuroimaging research, particularly fMRI studies, this review seeks to identify consistent patterns, highlight unresolved questions, and outline future research directions that may advance understanding of neuroimmune mechanisms in ASD and support the development of precision diagnostic and therapeutic strategies.

**Figure 1:**
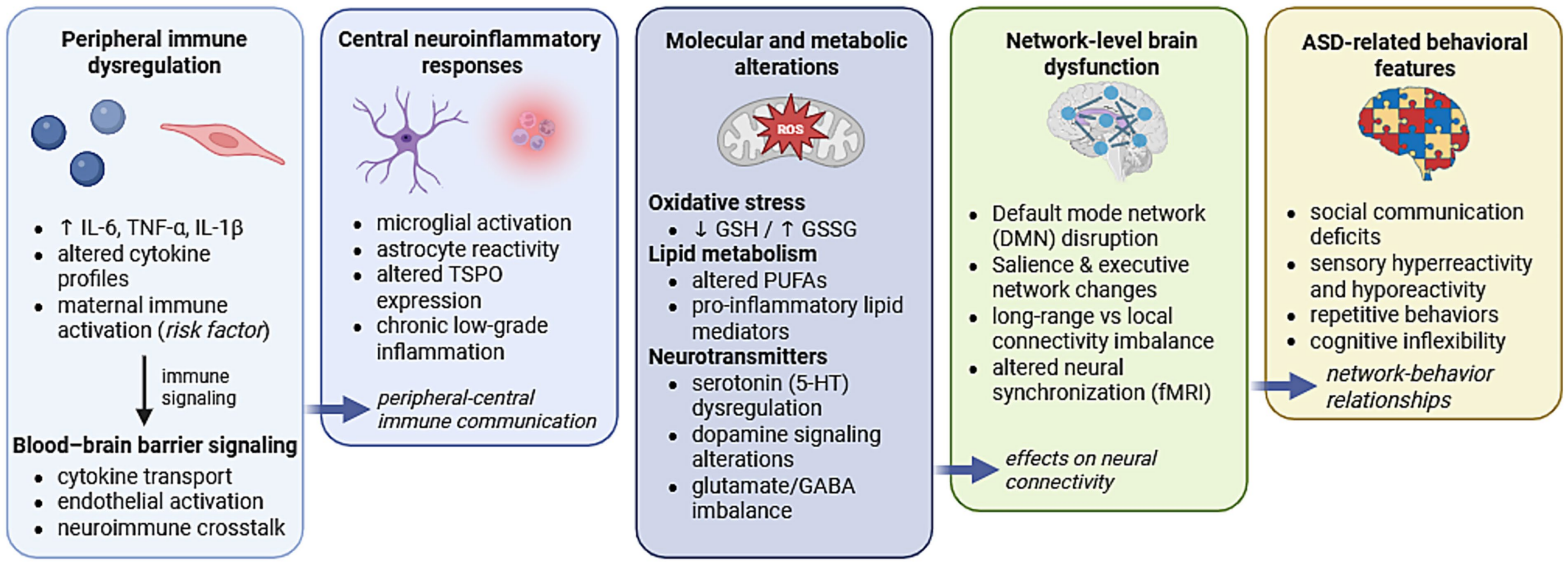
Multilevel interactions between neuroinflammatory processes and functional brain connectivity alterations in autism spectrum disorder. Arrows indicate proposed associations and modulatory interactions rather than direct causality. Created with https://www.biorender.com/.

Figure 1 presents an integrative multilevel model summarizing the mechanisms discussed in this review. In this framework, peripheral and central immune dysregulation characterized by elevated cytokines and glial activation initiates inflammatory signaling within the central nervous system. These immune processes promote oxidative stress, mitochondrial dysfunction, and disturbances in lipid and sulfur amino acid metabolism, thereby affecting neuronal redox balance and synaptic physiology. Such metabolic and inflammatory alterations can disrupt neurotransmitter systems, particularly glutamatergic, GABAergic, serotonergic, and dopaminergic signaling, leading to altered excitatory–inhibitory balance during neurodevelopment. At the systems level, these molecular and cellular disturbances may manifest as atypical functional connectivity within large-scale brain networks, including the default mode, salience, and executive control networks, ultimately contributing to the behavioral phenotype observed in ASD.

## Search strategy and study selection

This work represents a narrative review aimed at synthesizing multidisciplinary evidence rather than a formal systematic review. A comprehensive literature search was conducted using major full-text databases, including PubMed, Scopus, Web of Science, and Google Scholar. Both controlled vocabulary (e.g., MeSH terms in PubMed) and free-text keywords were used. The primary search terms included “autism spectrum disorder,” “*neuroinflammation*, “*cytokines*,” “*functional connectivity*,” and “*resting-state fMRI*.” Boolean operators (AND, OR) and combinations of related terms were applied to refine the results, for example: “autism AND neuroinflammation,” “microglia OR cytokine AND fMRI,” and “ASD AND functional connectivity AND immune response.” The search was adapted to each database to ensure comprehensive coverage and included English-language studies mostly published between 2010 and 2025. Titles and abstracts were screened for relevance, and eligible papers were those addressing the link between neuroinflammatory processes and functional brain alterations in individuals with ASD.

## Imaging techniques used in the assessment and characterization of ASD

Neuroimaging methods are valuable tools that support the diagnosis and assessment of ASD. These non-invasive approaches have enabled the confirmation of elevated levels of neuroinflammatory markers in the brain and cerebrospinal fluid (CSF) of individuals with ASD (Li et al., 2009; Edmonson et al., 2014). Positron emission tomography (PET) using ligands that bind to the translocator protein (TSPO), a marker of microglial activation, has revealed increased radiotracer uptake in the frontal cortex, cingulate gyrus, and cerebellum of individuals with ASD, suggesting the presence of ongoing neuroinflammatory processes (Suzuki et al., 2013; Hughes et al., 2023) and a potential association with the severity of social symptoms; however, interpretation of TSPO PET is complicated by genotype-dependent binding affinity and its lack of absolute specificity for microglial activation (Corrigan et al., 2013). Proton magnetic resonance spectroscopy (^1H-MRS) represents another tool that allows for the assessment of biochemical markers related to neuroinflammation. In individuals with ASD, elevated concentrations of myo-inositol, a metabolite associated with astroglial activity, have been reported in the frontal cortex and cingulate gyrus (Corrigan et al., 2013). Similar findings have also been observed for increased glutamate levels, which may reflect disturbances in excitatory neurotransmission and oxidative stress accompanying chronic inflammation (Brown et al., 2013; Demler et al., 2023). The integration of neuroimaging findings with immunological and genetic analyses indicates that neuroinflammation may modulate functional neuronal connectivity, particularly within networks involved in social cognition and the default mode network (DMN). Functional magnetic resonance imaging has played a pivotal role in elucidating these alterations (Harikumar et al., 2021). This convergence of evidence linking inflammatory processes to functional brain alterations represents a major direction in current research on the neurobiological mechanisms underlying ASD.

## Neuroinflammation and functional changes in fMRI studies

Resting-state fMRI studies have demonstrated that individuals with ASD exhibit altered connectivity within the default mode network (DMN), the salience network, and the executive control network (Assaf et al., 2010). Recent advances in neuroimaging indicate that functional connectivity should not be considered a static property of brain networks but rather a dynamic process characterized by continuous transitions between multiple connectivity states. Studies investigating functional connectivity dynamics (FCD) have shown that individuals with ASD exhibit altered switching behavior between connectivity states, reduced flexibility of network transitions, and prolonged dwell time in specific network configurations. These abnormalities are particularly evident in large-scale networks such as the salience network, default mode network, and frontoparietal network. The aberrant switching behaviors observed in individuals with ASD exhibit age-specific changes across development from childhood to adulthood (Li et al., 2025).

Interestingly, these connectivity alterations follow distinct developmental trajectories across large-scale brain networks. While disruptions in the salience network appear to persist across childhood, adolescence, and adulthood, abnormalities in the default mode network are more pronounced during childhood and adolescence, whereas alterations in the frontoparietal network seem to peak during adolescence, suggesting that this developmental stage may represent a critical period for the reorganization of cognitive control networks in ASD (Li et al., 2025). Adolescence may represent a critical period for functional reorganization of the frontoparietal network, and understanding these changes could inform more effective planning of therapy and cognitive support for individuals with ASD (Wendelken et al., 2016).

Importantly, the degree of these connectivity disruptions correlates with the severity of social and communicative symptoms, as well as with inflammatory biomarkers in serum and cerebrospinal fluid (Gotts et al., 2012). PET-MRI studies in autism spectrum disorder report regionally altered TSPO binding, for example, lower TSPO signal in the precuneus and posterior cingulate of young adult males with ASD (Zürcher et al., 2021), supporting the hypothesis that neuroinflammatory alterations detectable with TSPO-PET may contribute to DMN dysfunction observed in ASD (Simpson et al., 2022). These findings suggest that neuroinflammatory processes may modulate neuronal communication by affecting neurotransmitter metabolism, cerebral blood flow, and glial–neuronal interactions, as well as through disturbances in neurogenesis and synaptic plasticity (Morgan et al., 2010). Moreover, correlations between peripheral inflammatory markers and functional brain changes are a growing area of interest. In children with ASD, higher levels of TNF-α and IL-1β have been associated with abnormal connectivity within frontal–temporal and precuneus networks, suggesting a potential influence of cytokines on regions involved in social processing and empathy (Edmonson et al., 2014). Accumulating multimodal evidence indicates that neuroimmune activation co-occurs with neurometabolic disturbances and altered functional connectivity in ASD. In particular, several ^1H-MRS combined with rs-fMRI studies have linked regional glutamate/GABA imbalances to altered intrinsic connectivity of core networks such as the DMN (Kapogiannis et al., 2013; Ajram et al., 2017). Such observations support the view that abnormalities in brain connectivity in ASD may arise from complex interactions between immune signaling, glial activation, and large-scale neuronal networks (Yirmiya and Goshen, 2011).

## Markers of oxidative stress and inflammation

As illustrated in Figure 1, oxidative stress and metabolic dysregulation represent key intermediate mechanisms linking immune activation with functional brain network alterations. Autism spectrum disorder is increasingly associated with redox imbalance and oxidative stress, which play a central role in its pathophysiology and are closely linked to neuroinflammation (Table 1). A consistent finding is glutathione metabolism dysregulation, marked by significantly reduced levels of reduced glutathione (GSH), increased oxidized glutathione (GSSG), and a lowered GSH/GSSG ratio, indicating a shift toward a pro-oxidant state, and suggesting that glutathione-related biomarkers may hold potential for early ASD diagnosis (Chen et al., 2021; Bjørklund et al., 2020). These redox changes are accompanied by elevated lipid peroxidation products such as malondialdehyde (MDA) and F2-isoprostanes, which are considered the gold standard biomarker of lipid peroxidation due to their high specificity and reliability in reflecting oxidative damage to membrane lipids (Frustaci et al., 2012; Mohammedsaeed and Alharbi, 2025). Disruptions in transmethylation and transsulfuration pathways further compromise antioxidant capacity, with elevated homocysteine and S-adenosylhomocysteine, and reduced methionine, cysteine, and SAM levels, pointing to a weakened capacity for detoxification and antioxidant regeneration (Chen et al., 2021). Indika et al. (2021) proposed a sulfur amino acid metabotype in ASD that unifies these metabolic shifts with impaired methylation and mitochondrial dysfunction. Mitochondrial deficits exacerbate oxidative stress by impairing energy metabolism and increasing reactive oxygen species, sustaining a feedback loop of cellular damage (Rossignol and Frye, 2014). In parallel, chronic immune activation, particularly involving monocytes and macrophages, contributes to elevated cytokines and systemic inflammation, further interacting with redox pathways (Nour-Eldine et al., 2022; Liu et al., 2022). Deficiencies in antioxidant micronutrients such as vitamins B9, B12, D, E, and trace elements like selenium and calcium may further weaken antioxidant defenses and worsen neurodevelopmental outcomes (Mohammedsaeed and Alharbi, 2025).

**Table 1:** Oxidative stress–related biomarkers and redox alterations reported in autism spectrum disorder.

References	Population	Biomarkers assessed	Sample type	Key findings
Chen et al. (2021)	87 studies; 4,928 ASD and 4,181 controls	GSH, GSSG, tGSH, GSH/GSSG, MDA, homocysteine, SAH, vitamins D, B9–B12, Cu, Ca, NO	Elevated MDA and lipid peroxidation in cerebellum and temporal cortex	Significant redox imbalance: ↑ oxidative markers, ↓ antioxidants; glutathione biomarkers strongly differentiated ASD children
Bjørklund et al. (2020)	12 studies, various analyses	GSH, GSSG, GSH: GSSG ratio, GPx, inflammatory pathways (NF-κB, AP-1)	↓ GSH, ↑ 8-OHdG in prefrontal, orbitofrontal cortex	Glutathione system central in ASD pathology; redox imbalance impacts neurotransmission, inflammation, methylation
Mohammedsaeed and Alharbi (2025)	41 studies	Thiol/disulfide balance, GPx, SOD, catalase, brain oxidative damage markers	–	Confirmed impaired antioxidant enzyme activity and thiol/disulfide homeostasis in ASD children
Rossignol and Frye (2014)	Multiple studies; children and adults with ASD	GSH, GSSG, MDA, 8-OHdG, nitrotyrosine, SOD, GPx, mitochondrial enzymes	↓ GSH and antioxidant enzymes in cerebellum and prefrontal cortex; ↑ MDA, 8-OHdG, and nitrotyrosine in temporal and orbitofrontal cortex; mitochondrial dysfunction in brain tissues	↑ oxidative stress, ↓ antioxidant defenses, mitochondrial dysfunction, oxidative damage linked to symptom severity
Frustaci et al. (2012)	29 articles, ASD children and controls	GSH, GSSG, GPx, methionine, cysteine, SOD, homocysteine, cystathionine, vitamins A, B6, B12, C, D, E, ceruloplasmin, catalase, cysteinylglycine, TBARS, NO, MTHFR C677T polymorphism	–	↓ GSH, GPx, methionine, cysteine; ↑ GSSG; no change in SOD/homocysteine/cystathionine; MTHFR TT genotype increases ASD risk (OR 2.26)
Liu et al. (2022)	13 RCTs, 570 ASD (293 intervention, 277 placebo)	Antioxidants (mainly N-acetylcysteine)	–	Small, but significant improvements in irritability and stereotypy in intervention groups
Indika et al. (2021)	Mostly children with ASD, controls	Methionine, SAM, SAH, homocysteine, cysteine, cystathionine, taurine, cysteic acid, SAM/SAH ratio, GSH, cytokines	Postmortem ASD brains: oxidative stress, neuroinflammation, ↓ MS activity, ↓ cobalamin, ↓ PDH activity, mTOR signaling alterations	Methionine ↓, SAM ↓, SAH ↑, SAM/SAH ratio ↓, cysteine ↓, homocysteine ↑/↓ (inconsistent), GSH/GSSG ratio ↓, pro-inflammatory cytokines ↑, transsulfuration pathway ↑, methylation capacity ↓, melatonin ↓
Nour-Eldine et al. (2022)	61 studies; 2,875 ASD and 2,587 controls	42 cytokines including IL-1β, IL-6, IL-17, TNF-α, IL-4, IL-10, IFN-γ	Postmortem ASD brains: IL-6 ↑, IL-1β ↑, TNF-α ↑; neuroinflammation and immune activation observed	IL-1β ↑, IL-6 ↑, IL-17 ↑, TNF-α ↑, IFN-γ ↑, IL-12 ↑, IL-4 ↑, IL-10 ↑; consistent ↑ in pro-inflammatory and some anti-inflammatory cytokines, suggesting immune dysregulation in ASD

Immune system abnormalities have been reported in ASD by research groups worldwide. Immune dysregulation in ASD is characterized by neuroinflammation, the presence of autoantibodies, elevated T cell activity, and heightened innate immune responses involving natural killer (NK) cells and monocytes (Mead and Ashwood, 2015). Elevated peripheral inflammatory activity in ASD patients has been demonstrated through analyses of cytokine profiles. Numerous studies have shown altered cytokine levels in the blood, brain, and cerebrospinal fluid (CSF) of individuals with ASD compared to healthy controls. Both pro- and anti-inflammatory cytokines appear to play significant roles in the development of ASD (Table 2). Furthermore, abnormal cytokine profiles have been identified in brain tissue from individuals with ASD compared to healthy controls (Edmiston et al., 2017). Certain cytokines, such as IL-1α, IL-1β, IL-6, and TNF-α, can migrate from the periphery into the brain via blood–brain barrier (BBB) transport systems (Jiang et al., 2022). Cytokines and their receptors are normally present in the central nervous system, where they modulate neuronal differentiation and plasticity. An imbalance in cytokine levels can lead to chronic brain inflammation, known as neuroinflammation, which negatively affects neuronal development and activity, thereby impairing behavior (Bryn et al., 2017). Immune-related mechanisms influencing ASD risk may emerge as early as the prenatal period. Inflammation, infection, and stress during pregnancy may activate the maternal immune system and disrupt fetal development, and maternal autoimmune diseases beginning during pregnancy can strongly increase risk of ASD in offspring (Chen et al., 2016). Maternal immune activation (MIA) leads to abnormal synaptic pruning and connectivity, especially in regions critical for social behavior: prefrontal cortex, amygdala, hippocampus (Ayoub, 2025). Elevated concentrations of IFN-γ, IL-4, and IL-5 were observed in the serum of women at 15–19 weeks of gestation who later gave birth to a child diagnosed with ASD compared to women who gave birth to normal children (Goines et al., 2011). Immune alterations associated with ASD may also be detectable at birth and throughout early development. Peripheral cytokine profiles at birth have been associated with later ASD diagnosis and appear to vary based on ASD severity. Elevated IL-4 levels at birth were associated with a higher likelihood of severe ASD, whereas increased IL-1β levels were linked to milder forms of the disorder (Krakowiak et al., 2017). Elevated plasma levels of IL-1β, IL-6, IL-8, and IL-12p40 in children with ASD have been linked to more severe communication deficits and behavioral challenges, particularly in those with regressive autism (Ashwood et al., 2011). Moreover, Anastasescu et al. reported significantly higher TNF-α levels in children with ASD under the age of five compared with older children and control subjects. These elevated TNF-α concentrations in younger children may reflect a more pronounced inflammatory response during early development, potentially contributing to the onset and progression of ASD symptoms (Anastasescu et al., 2024). Growing evidence indicates that immune signaling plays an important role in shaping brain development during critical developmental periods. Cytokines and other immune mediators regulate processes such as neuronal proliferation, migration, and synaptic plasticity, which are essential for the maturation and organization of neural networks (Bilbo and Schwarz, 2012; Knuesel et al., 2014). During adolescence in particular, when large-scale brain networks undergo extensive reorganization, peripheral inflammatory signals have been associated with alterations in resting-state functional connectivity, including changes in amygdala–striatal and fronto-parietal circuits (Swartz et al., 2021). Brain networks supporting emotional regulation and social cognition continue to mature throughout adolescence, making them particularly sensitive to immune-related signaling during these critical developmental windows (Casey et al., 2019; Swartz et al., 2021). Taken together, these findings suggest that immune dysregulation occurring from the prenatal period through childhood and adolescence may influence the trajectory of brain network maturation of large-scale brain networks, potentially contributing to the atypical connectivity patterns observed in ASD.

**Table 2:** Peripheral and central inflammatory cytokine alterations in autism spectrum disorder.

References	Population	Markers	Key findings
Saghazadeh et al. (2019a)	25 studies; 1,022 ASD and 732 controls	Plasma and serum levels of 9 anti-inflammatory cytokines (IFN-α, IL-1Ra, IL 2Ra, IL-4, IL-5, IL-9, IL-10, IL-13, and TGF-β)	↓IL-10 and IL-1Ra, while IL-5 was slightly ↑ in ASD
Saghazadeh et al. (2019b)	38 studies; 1,393 ASD and 1,094 controls	Whole blood, plasma, and serum levels of IFN-γ, IL-1β, IL-6, TNF-α, TNF-β, IL-1α, IL-2, IL-2R, IL-7, IL-8, IL-12, IL-15, IL-17, IL-18, IL-23	↑IFN-γ, IL-1β, IL-6, TNF-α in ASD
Masi et al., 2015	17 studies: 743 ASD and 592 controls	Plasma and serum concentrations of 19 cytokines, chemokines and cytokine receptors (IL-4, IL-6, IL-8, IL-10, IL-12p40, IL-17, IL-23, IL-1α, IL-1β, IL-1RA, granulocyte colony-stimulating factor, TGF-β1, TNF-α, eotaxin, MCP-1; MIP-1α, MIP-1β, RANTES)	↑IL-1β, IL-6, IL-8, IFN-γ, eotaxin, and MCP-1,↓TGF-β1 in ASD
Zhao et al. (2021)	326 studies, ASD children and controls	Plasma and serum concentrations of IL-6, IL-1β, IL-12p70, MIF, eotaxin-1, MCP-1, IL-8, IL-7, IL-2, IL-12, TNF-α, IL-17, and IL-4	↑ IL-6, IL-1β, IL-7, IL-12p70 in ASD

## Lipid metabolism

Dysregulated lipid metabolism is a contributing factor in the pathophysiology of ASD, with alterations affecting both structural membrane lipids and signaling pathways (Table 3). ASD-specific elevations in VLDL-bound fatty acids (linoleic, oleic, and arachidonic acid) were found to correlate significantly with higher ADOS Social Affect scores, indicating greater severity of social interaction difficulties and suggesting a behavioral link to lipidomic imbalances (Usui et al., 2020). Increased levels of pro-inflammatory lipid mediators, including leukotrienes and prostaglandins, have also been reported in the plasma of individuals with ASD, indicating an inflammatory lipid signaling profile (El-Ansary and Al-Ayadhi, 2012). This is consistent with broader lipidomic analyses highlighting disturbances in glycerophospholipids, sphingolipids, and eicosanoids, which are believed to impact membrane fluidity, neuroinflammation, and synaptic function (El-Ansary et al., 2020). Although essential fatty acid (EFA) supplementation has been explored as a therapeutic strategy, the clinical evidence remains inconclusive due to inconsistent trial outcomes and methodological variability (Pancheva et al., 2023). At the molecular level, transcriptomic analyses of postmortem ASD brain tissue have revealed convergent downregulation of genes involved in lipid metabolism and neuronal signaling, reinforcing the central role of lipid homeostasis in ASD neurobiology (Voineagu et al., 2011).

**Table 3:** Lipidomic alterations and inflammatory lipid mediators associated with autism spectrum disorder.

References	Population/sample	Markers	Key findings
Usui et al. (2020)	30 ASD and 30 controls (lipidomics); 152 ASD and 122 controls (lipoproteins)	Omega-3 and -6 fatty acids, VLDL, ApoB	↑ omega-3/6 FAs correlated with better social scores; ↑ VLDL-bound fatty acids but ↓ ApoB linked to altered lipid profiles
El-Ansary and Al-Ayadhi (2012)	20 ASD vs. 19 control (males)	8-isoprostane, prostaglandin E2 (PGE2), cysteinyl leukotriene	↑ proinflammatory lipid mediators associated with ASD severity
Pancheva et al. (2023)	12 RCTs children with ASD	Omega-3 and omega-6 fatty acids supplementation; omega-6/omega-3 ratio	Mixed evidence, no recommendation for EFAs as stand-alone therapy
El-Ansary et al. (2020)	ASD children vs. controls	Dicarboxylic acids, oxysterols, PUFAs, acyl-carnitines, sphingolipids, lipid mediators	Altered lipid metabolism linked to oxidative stress, neuroinflammation, mitochondrial dysfunction, and neurotransmitter imbalance
Voineagu et al. (2011)	Postmortem ASD brain samples (19 ADS, 17 controls)	Gene expression of lipid metabolism pathways	↓ lipid biosynthesis and myelination gene expression

## Neurotransmitter imbalance

At the neurobiochemical level, brain activity and neural circuit function are modulated by a complex network of neurotransmitters. Growing evidence implicates disturbances in major neurotransmitter systems—particularly glutamatergic, GABAergic, serotonergic (5-HT), and dopaminergic signaling—in the pathophysiology of ASD (Jiang et al., 2022). Within the multilevel framework illustrated in Figure 1, neurotransmitter disturbances represent a key intermediate step through which immune and metabolic abnormalities may influence large-scale neuronal communication. Importantly, serotonergic and dopaminergic systems are highly sensitive to inflammatory signaling, providing a mechanistic link between immune dysregulation and neurotransmitter imbalance in ASD. Hyperserotonemia, characterized by elevated levels of serotonin (5-hydroxytryptamine or 5-HT) in the blood, was the first biomarker identified in autism research and remains one of the most consistently observed quantitative traits in a significant subset of individuals with autism spectrum disorder (ASD) (Gabriele et al., 2014). 5-HT regulates key developmental processes—including neurogenesis, neuronal maturation, innervation, synaptogenesis, and CNS signaling—culminating in behavioral outcomes. It also modulates immune functions such as T- and B-cell proliferation, inflammatory responses, and autoimmunity (Jaiswal et al., 2015). A systematic literature review and meta-analysis have shown that elevated blood 5-HT levels are currently the most consistently replicated biomarker of ASD, regardless of the biological matrix analyzed or the analytical method used (Gabriele et al., 2014). Other systematic analyses confirm that 5-HT levels are generally higher in individuals with ASD, supporting the concept of hyperserotonemia as an ASD endophenotype (Esposito et al., 2024). Genetic variants in SLC6A4, including the 5-HTTLPR polymorphism, have been repeatedly linked to serotonergic dysregulation in ASD. It is noteworthy that individuals with ASD have alterations in dopamine in the brain, whole blood, and urine (Lu et al., 2024). Substantial evidence indicates that dopamine (DA) plays a critical role in the establishment of typical brain structure and function. DA and its receptors are expressed in the striatum and frontal cortex during early neurodevelopment, where they contribute to the maturation of pathways underlying motor control, cognition, and reward processing. Alterations in DA signaling have been shown to influence the proliferation, migration, and differentiation of specific neuronal subpopulations, thereby impacting the organization of frontal cortical and striatal neurocircuitry (Money and Stanwood, 2013). Dysregulation of neurotransmitter transport and synaptic signal transduction has been implicated as an important contributor to ASD pathophysiology throughout different stages of neurodevelopment (Li et al., 2025).

## Genetic and epigenetic basis of ASD

The genetic architecture of ASD consists of a complex array of both rare (e.g., copy-number and single nucleotide variants, chromosomal abnormalities) and common single nucleotide polymorphisms acting additively to augment individual ASD risk (Grove et al., 2019; Pugsley et al., 2022). ASD is molecularly defined in about 20% of patients, and this category includes chromosomal alterations, including isodicentric 15q, ASD risk genes, and ASD-associated copy number variants (CNVs). However, about 75% of ASD is at present undefined (Satterstrom et al., 2020; Blum et al., 2024). Several genome-wide studies have been conducted to identify the genetic variants associated with risk for autism (Glessner et al., 2014; Hayretdag et al., 2020; Masini et al., 2020). CNVs, including several large recurrent deletions or duplications have been found. The most well-established autism-associated CNVs involve loci at 7q11.23, 15q11–13, 16p11.2, and 22q11.2, as well as genes such as NRXN1, CNTN4, NLGNs, and SHANK3 (Glessner et al., 2014). Whole-exome sequencing in twins with ASD has identified mutations in known ASD-related genes like FMN2, KCNQ2, NOTCH3, TMR6CA, SHANK3, and SLC6A4, reinforcing the genetic basis (Hayretdag et al., 2020). Recent studies have shown that epigenetic factors, including DNA methylation, histone modifications, and microRNAs (miRNAs), could play an important role in predisposition to autism (Masini et al., 2020). Several studies show that microRNAs (miRs/miRNAs) are strongly implicated in the development of ASD and affect the expression of genes related to different neurological pathways (Huang et al., 2015; Nguyen et al., 2016). A systematic review and meta-analysis identified miR-451a, miR-144-3p, miR-23b, miR-106b, miR-150-5p, miR-320a, miR-92a-2-5p, and miR-486-3p as the most consistently dysregulated miRNAs across studies, suggesting their potential as candidate biomarkers for ASD. Among these, miR-451a appears to be the most clinically relevant, as it is associated with impaired social interaction in individuals with ASD (Garrido-Torres et al., 2024).

Importantly, emerging evidence suggests that epigenetic mechanisms may also interact with immune and inflammatory signaling pathways implicated in ASD. Epigenetic modifications can regulate the expression of genes involved in cytokine signaling, microglial activation, and immune responses within the central nervous system. Transcriptomic analyses of postmortem ASD brain tissue have revealed coordinated dysregulation of genes related to synaptic function and immune processes, supporting the concept that genetic and epigenetic factors converge on neuroinflammatory pathways affecting brain development (Voineagu et al., 2011; Matta et al., 2019). Through these mechanisms, epigenetic regulation may represent a key interface linking genetic susceptibility with neuroimmune alterations that influence synaptic maturation, neuronal connectivity, and large-scale brain network organization in ASD (Masini et al., 2020; Herrera et al., 2024).

## Conclusion

Accumulating evidence indicates that specific inflammatory and oxidative stress-related markers detected in serum, cerebrospinal fluid, or brain tissue may potentially serve as informative biomarkers supporting the characterization and biological subtyping of autism spectrum disorder (ASD). Their assessment has the potential to facilitate identification of individuals at higher risk for pronounced social, sensory, and behavioral impairments, enable monitoring of disease progression or therapeutic response, and support the selection of treatment strategies tailored to an individual’s immunological and metabolic profile. Importantly, integrating inflammatory and redox biomarkers with functional neuroimaging measures, particularly fMRI and magnetic resonance spectroscopy, allows immunological alterations to be examined in direct relation to large-scale brain network organization and neurometabolic function. Such multimodal approaches offer a promising framework for developing more precise predictive and diagnostic models and for advancing personalized therapeutic strategies in ASD.

The findings summarized in this review suggest a multilevel biological cascade linking immune dysregulation with large-scale brain network dysfunction in autism spectrum disorder. Neuroimmune activation characterized by elevated cytokines and glial activation may promote oxidative stress and metabolic disturbances, including mitochondrial dysfunction and lipid dysregulation, which in turn affect neurotransmitter systems and synaptic homeostasis. These molecular and cellular alterations may ultimately manifest at the systems level as atypical functional connectivity within large-scale brain networks, contributing to core features of the ASD phenotype, including sensory disruptions, attentional difficulties, and aspects of social impairment. Nevertheless, current evidence remains largely correlational and heterogeneous, underscoring the need for cautious interpretation.

Future studies integrating longitudinal immune and metabolic profiling with multimodal neuroimaging will be critical for disentangling state-versus trait-related neuroinflammatory effects in ASD. Such approaches may also facilitate the identification of biologically defined ASD subgroups and advance the translation of neuroimmune and neuroimaging biomarkers into precision diagnostic and therapeutic frameworks. Large, well-characterized prospective cohorts will be essential to validate these markers and to clarify their relevance for clinical decision-making and intervention development.
